# [Expression of Concern] Interference of STAT 5b expression enhances the chemo-sensitivity of gastric cancer cells to gefitinib by promoting mitochondrial pathway-mediated cell apoptosis

**DOI:** 10.3892/or.2026.9075

**Published:** 2026-02-11

**Authors:** Tao Sun, Yanfei Jia, Dongjie Xiao

Oncol Rep 34: 227–234, 2015; DOI: 10.3892/or.2015.3994

Following the publication of the above paper, it was drawn to the Editor's attention by a concerned reader that the β-actin blots for the MGC-803 cell line in Fig. 4A were strikingly similar to the blots on the right-hand side of the gel intended to show the caspase-9 experiments in Fig. 7A; moreover, the blots shown for caspase-3 for the MGC-803 cell line in Fig. 4A were remarkably similar to blots that subsequently appeared in another article featuring one of the named authors (Tao Sun) in the same journal about a year later. In addition, a number of duplicated blots were noted comparing the flow cytometric plots in Figs. 3 and 6, including one blot which subsequently reappeared in a paper published in the journal *Molecular Medicine Reports* that was written by different authors.

The authors were contacted by the Editorial Office to offer an explanation for the apparent duplications of data both within the paper, and the subsequently published ones. Owing to the fact that the Editorial Office has been made aware of potential issues surrounding the scientific integrity of this paper, we are issuing an Expression of Concern to notify readers of this potential problem while the Editorial Office continues to investigate this matter further.

